# Onion Thrips, *Thrips tabaci*, Have Gut Bacteria That are Closely Related to the Symbionts of the Western Flower Thrips, *Frankliniella occidentalis*

**DOI:** 10.1673/031.008.2301

**Published:** 2008-03-19

**Authors:** Egbert J. de Vries, André W. G. van der Wurff, Gerrit Jacobs, Johannes A. J. Breeuwer

**Affiliations:** ^1^ Institute for Biodiversity and Ecosystem Dynamics, University of Amsterdam, Kruislaan 318, 1098 SM Amsterdam, the Netherlands.; ^2^ Laboratory of Nematology, Wageningen University, Binnenhaven 5, 6709 PD, Wageningen, the Netherlands

**Keywords:** symbiosis, 16S rDNA, bacterial taxonomy, *Erwinia*, *Pantoea agglomerans*, API 20E, insect gut

## Abstract

It has been shown that many insects have Enterobacteriaceae bacteria in their gut system. The western flower thrips, *Frankliniella occidentalis* Pergande [Thysanoptera: Thripidae], has a symbiotic relation with *Erwinia* species gut bacteria. To determine if other Thripidae species have similar bacterial symbionts, the onion thrips, *Thrips tabaci*, was studied because, like *F. occidentalis*, it is phytophagous. Contrary to *F. occidentalis, T. tabaci* is endemic in Europe and biotypes have been described. Bacteria were isolated from the majority of populations and biotypes of *T. tabaci* examined. Bacteria were present in high numbers in most individuals of the populations studied. Like *F. occidentalis, T. tabaci* contained one type of bacterium that clearly outnumbered all other types present in the gut. This bacterium was identified as an *Erwinia* species, as was also the case for *F. occidentalis*. However, its biochemical characteristics and 16S rDNA sequence differed from the bacteria present in *F. occidentalis*.

## Introduction

Bacteria are associated with a number of different insect species across all major orders of the Insecta (Buchner 1965; Campbell 1990; Dillon and Dillon 2004). Permanent associations between bacteria and higher organisms are called symbiotic and the type of symbiosis will depend on the particular host, the symbiont, and environmental conditions (Boucher et al. 1982; Douglas 1991; Bronstein 1994). For example, several Homoptera have a mutualistic symbiosis with *Buchnera* bacteria located in specific tissue near the gut, the so-called mycetomes. These mycetome bacteria provide the host with essential nutrients (Houk and Griffths 1980; Douglas 1989; Baumann et al. 1995). An example of parasitic symbiosis is the presence of *Wolbachia* bacteria in the reproductive tissue of many different arthropods. These bacteria interfere with host reproduction, which may lead to cytoplasmic incompatibility, parthenogenesis, or sex ratio distortion (Breeuwer 1997; Stouthamer et al. 1999; Vala 2003).

The insect gut provides a suitable habitat for bacteria (Bignell 1983). In many insect species the gut possesses different types of bacteria, which are transient and do not remain in the gut during all life stages. However, in some cases, a variety of permanent microorganisms is present that supply essential nutrients to their host (Blattidae, Cruden and Markovetz 1984; Termitidae, Breznak 1982; and Curculionidae, Bridges 1981). Ullman et al. 1989 were the first to detect gut bacteria in Thysanoptera in the hindgut of the western flower thrips, *Frankliniella occidentalis* Pergande [Thysanoptera: Thripidae]. These gut bacteria were isolated and characterised (De Vries et al. 1995 and 2001a). *F. occidentalis* were found to contain a single species of gut bacteria of the genus *Erwinia* (Enterobacteriaceae), possibly *Pantoea agglomerans*, which can also grow outside the insect host. Although several different growth media were tried to grow gut bacteria of these thrips, only one species of bacteria was consistently found. Based on 16S rDNA sequences derived from randomly selected bacterial colonies of *F. occidentalis*, only this type of bacteria was found, which adds further evidence to the conclusion that only one type of bacteria is consistently present in *F. occidentalis*. With the cloned16S rDNA fragments, universal primers were used to identify bacterial types present in the thrips regardless of whether they could be grown on bacterial medium (De Vries et al. 2001a). The *Erwinia* bacteria present in the thrips' gut are transmitted to progeny via the leaves that both adults and larvae eat (De Vries et al., 2001b). In second instar larvae, all thrips are infected with *Erwinia*, and up to 10^5^ bacteria are present per thrips. The presence of bacteria in *F. occidentalis* was found to cause a faster larval development and higher oviposition compared to thrips without bacteria, but this effect is dependent on the thrips diet (De Vries et al. 2004).

The microbiology of Thysanoptera is not widely studied. Apart from the *Erwinia* bacterial symbionts in *F. occidentalis*, the presence of *Pantoea ananatis* in the guts of the tobacco thrips, *F. fusca* (Gitaitis et al. 2003; Wells et al. 2002), and of bacteria in mycetomes of *Caudothrips buffai* (Bournier 1961) was reported. *Caudothrips buffai* is a mycophagous insect outside the large thrips family of Thripidae. Bournier did not find mycetomes in any of the species of Thripidae that he studied.

*Thrips tabaci*, was selected for the present study for the following reasons. It is, like *F. occidentalis*, a polyphagous pest insect. Contrary to *F. occidentalis*, it is endemic to Western Europe (Franssen and Mantel 1962). *T. tabaci* has a similar morphology and life cycle as *F. occidentalis* (Van Rijn et al. 1995). It is haplodiploid and arrhenotokous like most Thripidae, which implies that haploid, unfertilised eggs become males and diploid, fertilised eggs females. However, thelytoky (unfertilised diploid eggs, becoming females) was found in some *T. tabaci* populations (Murai 1990; Nault et al. 2006; Zawirska 1976).

Several populations of *T. tabaci* were examined for the presence of gut bacteria by looking for bacteria that would grow on enriched agar medium, and belonged to the family Enterobacteriaceae. The populations of *T. tabaci* that were studied originated from different geographical locations and host plants. They also differed in sex ratio and reproduction. Earlier, the same populations were found to be highly variable in their ability to transmit phytopathogenic tospoviruses (Wijkamp et al. 1995). Based on host plant, reproduction, and virus transmission ability, it may be argued that the *T. tabaci* populations studied in fact belonged to different biotypes as defined by Diehl and Bush (1984); populations belonging to the same species but differ in essential biological features.

**Table 1.**
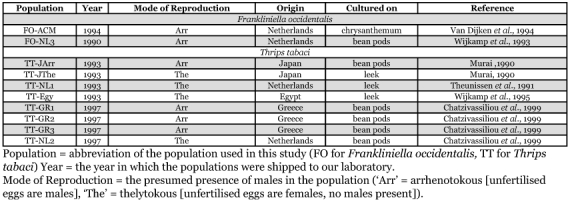
Populations of *Frankliniella occidentalis*, and *Thrips tabaci* used in this study.

## Material and Methods

### Thrips populations

*Thrips tabaci* populations used in this study were maintained at the Department of Virology of the Wageningen Agricultural University. These populations were originally obtained from various laboratory cultures (Table 1) and included thelytokous and arrhenotokous populations from different host plants. *T. tabaci* populations were given names with the abbreviation TT followed by country code and serial number, and *F. occidentalis* populations were abbreviated with FO (Table 1). One *F. occidentalis* population (FO-NL3) was reared on bean pods at the Department of Virology of the Wageningen Agricultural University. The other population of *F. occidentalis* (FO-AMC) was reared at the Institute for Biodiversity and Ecosystem Dynamics at the University of Amsterdam on chrysanthemum plants. Both *F. occidentalis* populations were originally collected in the Netherlands. Species identity was regularly checked, and we never encountered an infestation of other thrips species in these cultures (B. Vierbergen, personal communication).

### Thrips rearings

Thrips were reared in small closed jars, 85 mm wide and 70 mm high, in climate rooms of 25° C, 60% RH, and 16:8 L:D. Pods of the bean, *Phaseolus vulgare*, were used as food for some populations (TT-JArr, TT-NL2 and the Greek populations). Leaf pieces of the leek, *Allium porrum*, were used for the other three populations (TT-JThe, TT-NL1, and TT-Egy). Bean pods and leek leaves were obtained from local groceries, washed, and incubated for four days at 25 °C. After being thoroughly washed again, these were used in the cultures. This procedure minimises the risk of contamination with other thrips. The *F. occidentalis* population FO-NL3 was reared in the same way. The FO-AMC population was reared on chrysanthemum plants in a climate box (25 °C, 60% RH, 16:8 L:D). Chrysanthemum plants were obtained from local garden shops and not washed.

### Isolation of bacteria

Samples of 20 to 30 thrips were selected and subjected to isolation of bacteria, following the protocol described earlier (De Vries et al. 2001a). Briefly, the thrips were placed individually in small vials (0.5 ml) and surface sterilised to avoid picking up external bacteria. Surface sterilisation is done by soaking individual thrips in 70% ethanol for 60 seconds, soaking it in a 5% sodium chlorite solution, and subsequently rinsing three times with sterilised water. Thrips were then homogenised in 100 µl Tris/EDTA buffer (10 mM Tris and 1 mM EDTA, pH = 7.6). Thrips homogenate was spread out on Luria-Bertoni (LB) agar medium and incubated for 48 h at 25 ° C. LB medium is the media on which gut bacteria of *F. occidentalis* were found to grow best (De Vries et al. 2001a). Earlier, ten different rich growth media were used for isolation of bacteria from thrips. This range of growth media fully covered of nearly all media that have been used for isolation of insect gut bacteria according to an extensive literature search (see De Vries et al. 2001a for a complete description). All steps of the bacterial isolation procedure took place in a laminar flow hood to reduce the chance of contamination. In 1995, isolations were done in Amsterdam at the Institute for Biodiversity and Ecosystem Dynamics, and in 1998 they were done at the Department of Virology in the Wageningen Agricultural University.

### Identification of bacteria

Only thrips homogenate that yielded more than 30 colonies were used, because less than 30 colonies could easily have resulted from accidental contamination during the bacterial isolation procedure. The prevalence was determined as the frequency of thrips with gut bacteria out of all thrips included in the experiment. Three methods were used to describe the gut bacteria. First, the morphology of the most frequent type of colonies in each thrips homogenate was recorded. The size was determined as small (< 3mm after 48 h of incubation at 25°C) or large. The colour was determined, as well as the shape (round or fuzzy) and the clarity (clear or opaque). Nearly all colonies obtained in our study were small, round and opaque. Second, pure strains were obtained of this type of bacteria by transferring a single colony to fresh LB agar medium twice. These pure strains were biochemically identified using the API 20E method (Biomérieux; http://www.biomerieux.com). This method is a standardised test for the following twenty biochemical characteristics; beta-galactosidase, acetoin production, cytochrome oxidase, arginine dehydrolase, lysine dehydrolase, ornithine dehydrolase, H2S production, urease, tryptophan deaminase, indole production, gelatinase, and the utilisation of glucose, mannitol, rhamnose, sucrose, amygdalin, arabinose, inositol, sorbitol, and citrate. The biochemical characteristics according to the API 20E system were compared with characteristics of two type strains of *F. occidentalis* gut bacteria, TAC XII.93.8 and TAC III.94.1 (described in De Vries et al. 2001a, and determined again in this study). The third identification method involved determining the 16S rDNA sequence of a few pure strains of *T. tabaci* gut bacteria and comparing them with sequences of gut bacteria from *F. occidentalis*, and with other Enterobacteriaceae.

### 16S rDNA sequence comparison

DNA was isolated from bacteria using a method based on Chelex 100 (Walsh et al. 1991). A colony of a pure strain of bacteria was suspended in an emulsion of 1ml 5% (w/v) Chelex 100 (Sigma-Aldrich, www.sigmaaldrich.com) in sterile tissue water and 20 µl proteinase K (20 mg/ml) was added per DNA sample. Samples were incubated for 2 h at 37 °C and after this, proteinase K was denatured during 15 min at 95 ° C. The obtained DNA isolate was used for 16S rDNA amplification. Part of the 16S rDNA gene was amplified using universal bacterial primers (Lane 1991), based on the *Escherichia coli* genome (27F and 1492R) by PCR with cycle conditions as described elsewhere (De Vries et al. 2001a). The PCR product was run on a 1.0% (w/v) agarose gel, and the 16S rDNA gene, of approximately 1500 bp, was excised from the gel, purified and sequenced on a LiCor automated DNA-sequencer using dye-primer chemistry following the manufacturers protocol (BaseClear Ltd., www.baseclear.com).

The resulting 16S rDNA sequences were manually aligned and compiled in one database. Several 16S rDNA sequences of other Enterobacteriaceae were taken from the GenBank and included in the database: *Erwinia herbicola, E. coli, Citrobacter freundii, Erwinia carotovera, Serratia marcescens*, and *Proteus vulgaris*. Sequences were also determined of *Erwinia* sp. gut bacteria from *F. occidentalis* that had been isolated in 1994 and kept in the -70°C freezer (see De Vries et al. 2001a, for a description of the bacteria isolation). These sequences were also included in the database. The aphid mycetome symbiont, *Buchnera aphidicola,* a non-Enterobacteriaceae species belonging to the gamma-Proteobacteriae, was used as the outgroup. The database was subjected to parsimony analysis using Paup 3.1.1 (Swofford 1990). Gaps were treated as missing data. A phylogram was created using the heuristic search option with 1000 fold bootstrapping. The 16S rDNA sequences from *T. tabaci* and *F. occidentalis* gut bacteria determined in this study were deposited in the GenBank database at accession numbers AY616165 to AY616181.

## Results

### Morphological characteristics of gut bacteria from onion thrips

In 1995, adult thrips were collected from four *T. tabaci* populations and one *F. occidentalis* population (FO-AMC) and gut bacteria were isolated from them (Table 2). The populations had then been maintained in the laboratory for more than one year. The *T. tabaci* populations showed large individual variation in the presence of gut bacteria, ranging from no gut bacteria to many. *F. occidentalis* always had large numbers of bacteria per individual thrips in all populations that were tested (De Vries et al. 2001a). In the TT-JArr population, more than 50% of *T. tabaci* carried gut bacteria, whereas *T. tabaci* of the TT-NL1 and TT-Egy populations were usually without gut bacteria. One *T. tabaci* population, TT-JThe, could not be tested because the population was too small. The presence of gut bacteria in *T. tabaci* was independent of host plant and mode of reproduction, although the number of populations sampled was low.

**Table 2.**
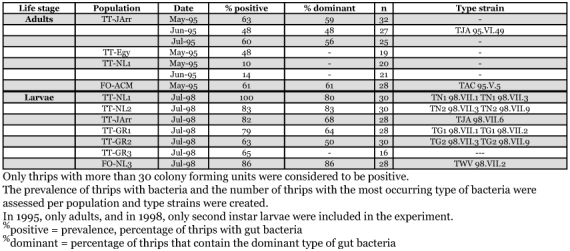
Presence of gut bacteria in *Thrips tabaci* populations, measured in 1995 and 1998.

Several different colony phenotypes were found in thrips from the TT-JArr *T. tabaci* population, but one type of colony was present in every positive thrips and its prevalence ranged from 48 to 59% of all the colonies (Table 2). A pure strain of bacteria with this particular colony phenotype was obtained and used as type strain for the gut bacteria in the TT-JArr *T. tabaci* population (TJA 95.VI.49). The *F. occidentalis* population showed the same pattern as had been consistently found previously in this thrips species: one dominant type of bacteria present in the majority of the adult insects (54%). The colonies, found in the *F. occidentalis* population, were phenotypically similar to the type strain of *Erwinia* species gut bacteria from this thrips species, TAC 93.XII.8 (De Vries et al. 2001a). One pure strain of the dominant bacterial type was obtained from gut bacteria isolated from *F. occidentalis* in this experiment, labelled as TAC 95.V.5, for comparison with TAC 93.XII.8. Interestingly, the dominant type of bacteria in *T. tabaci* population, TT-Jarr, also had the same morphological features as the *Erwinia* species gut bacterium TAC 93.XII.8 from *F. occidentalis*.

Three years later, bacteria were isolated again, but this time from second instar larvae, because they were expected to have the highest prevalence of bacteria (De Vries et al. 2001b). Indeed, five out of six populations of *T. tabaci* had a much higher prevalence of gut bacteria, ranging from 80% to 100% (Table 2). Only TT-GR3 had a low prevalence of 25%. The *F. occidentalis* population (FO-NL3) showed a high prevalence (86%) of gut bacteria, and the colonies were phenotypically similar to type strain TAC 93.XII.8. Again one dominant bacterial colony type was found in all or most individuals in each population of *T. tabaci*. In addition, various other phenotypes of colonies were found, but they were present in small numbers and not in all individuals. Again, the dominant phenotype had similar morphological characters, independent of the *T. tabaci* population, and resembled the *F. occidentalis* type strain TAC 93.XII.8 closely. To study the gut bacteria of *T. tabaci* in detail, pure bacterial strains were obtained from the dominant colony phenotype of each population. These strains were given names that reflected the name of the thrips population, the isolation date, and the thrips individual from which they had been isolated (Table 2). Additionally, a pure strain of bacteria was obtained from the gut bacteria of one of the larvae of the FO-NL3 *F. occidentalis* (strain TWV 98.VII.2).

### Biochemical identification

The biochemical identification of bacterial type strains was done using the API 20E system. This method was used earlier to typify the bacterial strains TAC 93.XII.8 and TAC 94.III.1 from the *F. occidentalis* population AMC as *Erwinia* species, possibly *Pantoea agglomerans*. The biochemical characteristics of both strains of *F. occidentalis* gut bacteria obtained in this experiment, TAC 95.V.5 and TWV 98.VII.2, were identical, and similar to the bacteria isolated before from this thrips species (Table 3). *F. occidentalis* gut bacteria were positive in beta-galactosidase, acetoin production, and the utilization of glucose, mannitol t, rhamnose, sucrose, amygdalin, and arabinose. They were negative for arginine dehydrolase, lysine dehydrolase, ornithine dehydrolase, citrate utilization, H2S production, urease, tryptophan deaminase, indole production, gelatinase, inositol utilization, sorbitol utilization, and cytochrome oxidase (code 1005373 in Table 3). The complete similarity between this and previous results (De Vries et al. 2001a) confirms that this bacterial strain is a persistent and dominant inhabitant of the gut of the *F. occidentalis*.

**Table 3.**
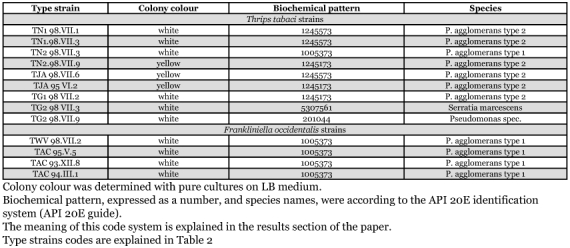
Biochemical pattern of type strains of gut bacteria from populations of *T. tabaci* and *F. occidentalis.*

The bacterial type strains from four out of five *T. tabaci* populations were similar to each other and also belonged to the genus *Erwinia* (TT-JArr, TT-GR1, TT-NL1, and TT-NL2). They were tentatively named *Pantoea agglomerans*: their biochemical characteristics were the same as those of the *F. occidentalis* gut bacteria, except that *T. tabaci* bacteria were positive in citrate utilization and indole production, and negative in inositol utilisation (Table 3). The type strains of *T. tabaci* gut bacteria, TN1 98.VII.1, TN1 98.VII.3, TN2 98.VII.9, TJA 98.VII.6, TJA 95.VI.2, and TG1 98.VII.3 were all completely similar except that some were able to utilise sorbitol and others were not (the difference between code 1245573 and 1245173 in Table 4, respectively). The gut bacteria type strains obtained from *T. tabaci* of the TT-GR2 population were completely different. One type strain, TG2 98.VII.3, was identified as a *Serratia marcescens*, and the other strain, TG2 98.YII.9, was a *Pseudomonas* species (Table 3).

### DNA relatedness between thrips gut bacteria

The final 16s rDNA dataset consisted of 26 entries of at least 1430 bp positions. All thrips gut bacterial strains showed at least 93 per cent sequence similarity to *E. coli*, indicating that they were Enterobacteriaceae. The only exception was strain TG2 98.VII.9, that was more related to *Pseudomonas* species, which was already indicated by the above API 20E tests. This strain was left out of the database.

**Figure 1.**
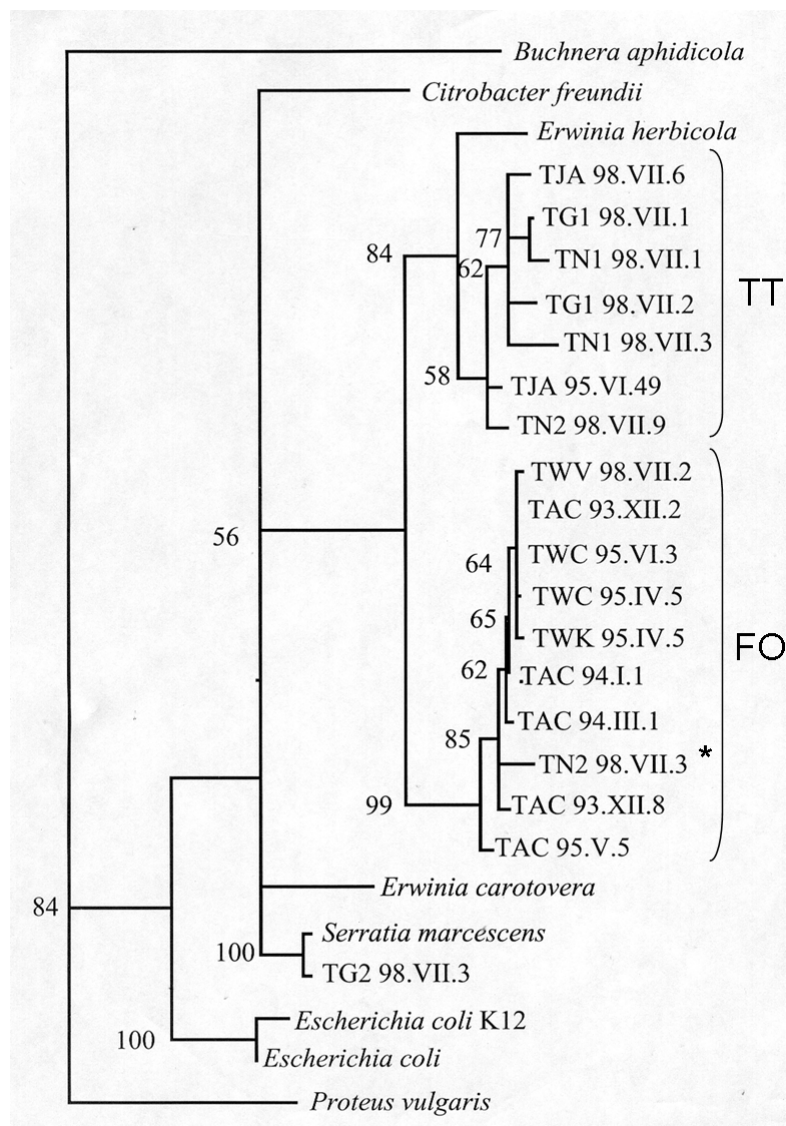
The consensus phylogenetic tree of the 16S rDNA gene of gut bacteria from populations of *Frankliniella occidentalis, Thrips tabaci*, and of several other species of Enterobacteriacea. The tree is based on at least 1430 bp per sequence and created using parsimony analysis. *Buchnera aphidicola* was used as outgroup. Bootstrap values over 1000 replicates are mentioned in front of the branches. The names of the type strains of gut bacteria are explained in Table 2. FO indicates the group of DNA sequences belonging to bacteria isolated from *Frankliniella occidentalis*, TT indicates the DNA samples isolated from bacteria found in *Thrips tabaci*. The DNA sequence labelled with an asterisk is the only sequence from a *Thrips tabaci* bacterium which was found in the FO group.

After parsimony analysis and bootstrapping, the shortest tree was selected (Figure 1). The phylogenetic positioning of thrips gut bacteria within the Enterobacteriaceae and their close relationship to *Erwinia herbicola,* confirms the above biochemical API identification. Two clades were observed and both were supported by high bootstrap values. One clade, labelled FO, comprised all *F. occidentalis* bacterial type strains. Gut bacteria from *F. occidentalis* isolated in the present study were highly similar to gut bacteria isolated from this insect species in earlier studies and activated again from frozen stock cultures (De Vries et al. 2001a). The other clade, the TT clade, comprised only *T. tabaci* bacterial type strains. The sequence difference between these two clades ranged from 94 to 96% similarity. Sequence similarity within clade FO and clade TT was 97% or higher. Two exceptions were found in the phylogeny of *T. tabaci* gut bacteria: one type strain (TN2 98.VII.3 of the TT-NL2 population) was placed within the monophyletic FO clade (indicated with an asterisk in Figure 1), and one type strain (TG2 98.VII.3 from population TT-GR2) clusters with a strain of *Serratia marcescens*, confirming the API 20E identification.

## Discussion

Associations of Thripidae insects with one particular strain of bacteria in their gut is a not a unique phenomenon. It was found earlier in *F. occidentalis* populations (De Vries et al. 2001a) and *F. fusca* (Wells et al. 2002). In this study the association of *T. tabaci* from different populations with gut bacteria is described. The thrips of these populations contain large amounts of bacteria in their gut, and one particular type of bacteria is dominant. Like *F. occidentalis* larvae, individual *T. tabaci* have up to 105 bacteria in second instar larvae and adults. Although experiments to isolate bacteria directly from the dissected gut have been done on *F. occidentalis*, but not on *T. tabaci*, the results obtained here strongly suggest that *T. tabaci* bacteria are also located in the thrips hindgut. Bacteria are probably taken up by young *T. tabaci*, settle in the hindgut and grow to high numbers while the insect host is feeding (see De Vries et al. 2001b). These results, combined with the study on *F. fasca* (Wells et al. 2002), suggest that gut bacteria may be widespread among *Thrips* and *Frankliniella* species.

Contrary to our previous study on *F. occidentalis*, different media were not used for bacterial growth from *T. tabaci*, nor has bacterial DNA, such as 16S rDNA, been directly obtained from a crude extract of *T. tabaci*. It is likely, therefore, that we do not have a full picture of *T. tabaci* microflora. It is possible that other opportunistic and facultative heterotrophic bacteria are present in *T. tabaci* that do not grow on the selected rich growth medium for bacteria (in this case LB medium). It is possible that unculturable, obligate symbiotic bacteria are present that are not able to grow outside the thrips gut. The previous study with *F. occidentalis*, confirmed that the type of bacterium dominantly present on bacterial growth medium after bacterial isolation from thrips was similar to the type of bacterium obtained with direct sequencing on crude DNA extract from thrips (De Vries et al. 2001b). Nault et al. (2006) tried to isolate *Wolbachia* bacteria from *T. tabaci* but did not find these endosymionts in this thrips species.

### Prevalence of gut bacteria in *T. tabaci*

The number of *T. tabaci* infected with bacteria within one population (the prevalence of bacteria) was similar to *F. occidentalis*. About 50% of the adults are infected with bacteria, but among larvae the prevalence was higher, a pattern that was also observed in *F. occidentalis* (De Vries et al. 2001b). In contrast to *F. occidentalis*, prevalence in *T. tabaci* larvae never reached one hundred per cent. This could be due to life stage-dependent infection prevalence. In *F. occidentalis* the highest prevalence of bacteria was always found in second instar larvae of a certain age. In this study, random samples of *T. tabaci* and *F. occidentalis* second instar larvae from the mass culture were used. Possibly, the reduced prevalence in both the FO and TT populations is due to the inclusion of younger thrips larvae.

### Variation in gut bacteria within a single thrips

Another difference between *T. tabaci* and *F. occidentalis* was the variation of gut bacteria in individual *T. tabaci*. There appeared to be variation in bacterial strains even in later stages of the *T. tabaci* life cycle, namely second instar larvae and adults. In *F. occidentalis* it was found that a variable gut microflora only occurs in first instar larvae. After this life stage the *Erwinia* species gut bacteria became dominant and also the only type of bacteria present in *F. occidentalis* (De Vries et al., 2001b). This was only found in the *T. tabaci* population TT-NL1. Unfortunately, it was not possible to repeat bacteria isolation experiments for all *T. tabaci* populations, because some of them were not maintained for three years in the laboratory, so it is not possible to compare data from different generations of thrips. Comparison was possible for some populations of *T. tabaci* such as TT-JArr, and it was found that the presence of bacteria is stable over many generations, as it was in *F. occidentalis*. In general it is important to study insects for a several generations before one can be certain about the exact nature of their symbiotic association with gut bacteria (De Vries et al. 2001a).

### *Erwinia* species gut bacteria

All *F. occidentalis* populations, and most *T. tabaci* populations studied, possess gut bacteria belonging to the genus *Erwinia*. The tentative species name of all bacterial type strains is *P. agglomerans*, which is based on biochemical identification (API 20E). The close relatedness of strains of bacteria from various *F. occidentalis* populations is confirmed by the 16S rDNA study (Figure 1). Despite the limited amount of 16S rDNA sequence differences between *F. occidentalis* and *T. tabaci* gut bacteria, a pattern seems to emerge that shows separation of FO and TT bacteria in two different clades. The *P. agglomerans* strains from nearly all *T. tabaci* populations cluster together in the TT clade, and all *P. agglomerans* strains from *F. occidentalis* cluster together in the FO clade.

In the past, the species names *Erwinia herbicola* and *P. agglomerans* have been used interchangeably (Mergaert et al. 1984). Our 16S rDNA study suggests that the types of *P. agglomerans* in the FO clade and in the TT clade are genetically more different (at most 96% similarity), but less different among each other (at least 97 % similarity). Only one clade (the TT clade) of *P. agglomerans* is related to the type strain of *E. herbicola* from the GenBank. *E. herbicola* bacteria have been found in other insect species. In their studies, Takahashi et al. 1995 and Watanabe *et al*. 1996 showed that this bacterial species is present in brown planthoppers, mulberry pyralids, and silkworms, and originate from their host plants. Other researchers have described associations of insect species and *Enterobacter agglomerans* (in *Psila*, Cole et al., 1990; locusts, Dillon et al. 2000; and Mead et al., 1988; fruit flies, MacCollem *et al*. 1992; bark beetles, Bridges 1981; termites, Potrikus and Breznak 1977; mosquitoes, Straif et al., 1998). *Enterobacter agglomerans* was renamed to *P. agglomerans* (Gavini et al. 1989). DNA sequence comparison is necessary to make any statement on the relatedness between gut bacteria from any of these insect species and from thrips.

### Bacteria not present in all onion thrips populations

Three out of eight *T. tabaci* populations had no internal bacteria or too few individuals with enough bacteria to yield a type strain of gut bacteria. One population had gut bacteria but they were quite diverse and unrelated. This result is in contrast to the observation that all *F. occidentalis* populations have gut bacteria. We have studied many populations of *F. occidentalis* (De Vries, unpublished results). Perhaps it is possible to classify *T. tabaci* populations as different biotypes depending on the presence or absence of gut bacteria. Diehl and Bush (1984) defined insect biotypes as populations of a polyphagous insect species that are related to different host plants. But the biotype concept is in principle applicable to all sorts of important insect attributions. For example, in other studies *F. occidentalis* biotypes were described regarding insecticide resistance (Brødsgaard 1994; Immaraju et al. 1992). *F. occidentalis* biotypes based on host plant utilisation were never found. *F. occidentalis* was only recently introduced to Europe, Asia and Africa (Brødsgaard 1994), and the thrips has a very broad host range (Yudin et al. 1986). In contrast, *T. tabaci* are endemic in most European countries. This would have given *T. tabaci* more time to develop host plant biotypes. Interestingly, the populations of *T. tabaci* cultured on leek were all without a type strain of gut bacteria, whereas the *T. tabaci* in which gut bacteria were found were cultured on bean pods. The possibility of a host plant effect in the transmission of gut bacteria should be taken into account. We have never found any host plant effect in bacteria transmission by *F. occidentalis*, but leek or any other member of the family Liliaceae was not included in these studies (De Vries et al. 2001b).

### Thrips-gut bacteria interaction

The presence of gut bacteria inside *T. tabaci* leads to the question what kind of interaction may take place between symbiont and host. The interaction between gut bacteria and *F. occidentalis* can be mutualistic depending on the thrips diet (De Vries et al., 2004). It was found that thrips with gut bacteria have a shorter larval development time and higher daily egg production when living on leaf discs, but on a diet of leaf discs and pollen this mutualistic effect of the presence of bacteria was not found. However, this cannot be directly extended to the *T. tabaci* — gut bacteria interaction, because *T. tabaci* have other diets, and other types of bacteria. For example, *T. tabaci* does not have a preference for pollen as is found in *F. occidentalis* (Van Rijn et al. 1995). In addition, much more variation in number and type of gut bacteria was found in *T. tabaci* compared to the *F. occidentalis*. Perhaps the presence of bacteria in *T. tabaci* is more commensalistic, where bacteria require the insects to spread to new host plants. This type of interaction was assumed for the planthoppers (Watanabe *et al.* 1996), and for *F. fusca* (Gitaitis et al. 2003).

Interestingly, Wijkamp et al. 1995 studied various populations of *F. occidentalis* and *T. tabaci* and found that there is also a large variation in the *T. tabaci* ability to transmit plant viruses. All *F. occidentalis* populations reliably transmit virus, although variation exists in transmission efficiency. Only one *T. tabaci* population, TT-JArr, successfully transmitted tomato spotted wilt virus, and other populations, TT-JThe, TT-Egy, and TT-NL1 did not. Of these populations, TT-JArr and TT-NL1 contain gut bacteria and TT-Egy does not. It is presently too speculative to hypothesize on the influence of gut bacteria on virus transmission by *T. tabaci.*
